# *Helicobacter pylori* infection is not associated with fatty liver disease including non-alcoholic fatty liver disease: a large-scale cross-sectional study in Japan

**DOI:** 10.1186/s12876-015-0247-9

**Published:** 2015-02-19

**Authors:** Kazuya Okushin, Yu Takahashi, Nobutake Yamamichi, Takeshi Shimamoto, Kenichiro Enooku, Hidetaka Fujinaga, Takeya Tsutsumi, Yoshizumi Shintani, Yoshiki Sakaguchi, Satoshi Ono, Shinya Kodashima, Mitsuhiro Fujishiro, Kyoji Moriya, Hiroshi Yotsuyanagi, Toru Mitsushima, Kazuhiko Koike

**Affiliations:** 1 Department of Gastroenterology, Graduate School of Medicine, The University of Tokyo, Tokyo, Japan; 2 Kameda Medical Center Makuhari (CD-2, 1–3, Nakase, Mihama-ku, Chiba-city, Japan

**Keywords:** Fatty liver disease, NAFLD, Metabolic syndrome, *Helicobacter pylori*

## Abstract

**Background:**

Fatty liver disease (FLD) including non-alcoholic fatty liver disease (NAFLD), a rapidly emerging and widely recognized liver disease today, is regarded as a hepatic manifestation of metabolic syndrome. *Helicobacter pylori*, one of the most common pathogens worldwide, has been reported to be associated with metabolic syndrome, but whether there is a direct association with FLD is as of yet unclear. The aim of this study was to clarify the association of FLD and NAFLD with causative background factors including *Helicobacter pylori* infection.

**Methods:**

This was a cross-sectional study of Japanese adults who received medical checkups at a single medical center in 2010.Univariate and multivariate statistical analysis was performed to evaluate background factors for ultrasonography diagnosed FLD. Subjects free from alcohol influence were similarly analyzed for NAFLD.

**Results:**

Of a total of 13,737 subjects, FLD was detected in 1,456 of 6,318 females (23.0 %) and 3,498 of 7,419 males (47.1%). Multivariable analyses revealed that body mass index (standardized coefficients of females and males (β-F/M) =143.5/102.5), serum ALT (β-F/M = 25.8/75.7), age (β-F/M = 34.3/17.2), and platelet count (β-F/M = 17.8/15.2) were positively associated with FLD in both genders. Of the 5,289 subjects free from alcohol influence, NAFLD was detected in 881 of 3,473 females (25.4%) and 921 of 1,816 males (50.7%). Body mass index (β-F/M = 113.3/55.3), serum ALT (β-F/M = 21.6/53.8), and platelet count (β-F/M = 13.8/11.8) were positively associated with NAFLD in both genders. Metabolic syndrome was positively associated with FLD and NAFLD only in males. In contrast, *Helicobacter pylori* infection status was neither associated with FLD nor NAFLD regardless of gender.

**Conclusions:**

Body mass index, serum ALT and platelet count were significantly associated with FLD and NAFLD, whereas infection of *Helicobacter pylori* was not.

**Electronic supplementary material:**

The online version of this article (doi:10.1186/s12876-015-0247-9) contains supplementary material, which is available to authorized users.

## Background

Fatty liver disease (FLD) is the most common chronic liver disease in the world today. It is caused by multiple factors such as nutritional disorders, dyslipidemia, insulin resistance, genetic factors, etc. [1]. Especially, alcohol intake and metabolic abnormalities such as insulin resistance have been reported to be the main causes for FLD [2,3]. However, recent epidemiological studies have not been enough to elucidate complicated risk factors for FLD, despite the high prevalence and importance of the disease [4-6]. FLD is generally divided into alcoholic fatty liver disease (AFLD) and non-alcoholic fatty liver disease (NAFLD) according to amounts of alcohol intake. The boundary value of alcohol intake between AFLD and NAFLD is tentatively defined [7], but the effect of moderate alcohol intake upon FLD still leaves much room for discussion [8-10].

NAFLD is also common all over the world including Eastern countries [11], but the reported prevalence rate of NAFLD varies widely [12-17]. NAFLD is a concerning disease not only because of its high prevalence but also its potential risk of fatal diseases such as liver failure, hepatocellular carcinoma (HCC), cardiovascular disease, and so on [18,19]. NAFLD is regarded as a hepatic manifestation of metabolic syndrome (MS) [20]. Machado M *et al*. reported that the prevalence of NAFLD was 91% in obese patients who had undergone bariatric surgery [21]. It has also been reported that type 2 diabetes and other features of MS are strongly related to NAFLD [22,23].

The relationship between NAFLD and microbes in the gut has been occasionally reported [24,25]. As ”Gut-Liver Axis” has been widely noticed [26,27], several liver diseases including NAFLD are thought to be influenced by gastro-intestinal tract environments mainly decided by existing microbes. Among enormously varied microbes, to our knowledge today, *Helicobacter pylori* (*H. pylori*) shows the greatest effect on the upper gastro-intestinal environment. It is well known that approximately 50% of the global population is estimated to be infected by *H. pylori* [28], and is also well established that chronic infection of *H. pylori* can be a cause of chronic atrophic gastritis, peptic ulcer disease and gastric cancer [29,30]. Recently, not a few reports concerning the influence of *H. pylori* on various extra-alimentary organs have been accumulated [31-34]. Among these putative extra-alimentary disorders caused by *H. pylori*, the relation to MS is still controversial[35-42]. Though there have been many reports discussing the relationship between *H. pylori* and MS, issues to clarify still remain.

Based on these reports, we hypothesized that *H. pylori* has some associations with FLD including NAFLD. There has previously been only one similar small-scale study which concluded that *H. pylori* infection is associated with NAFLD [43]. The aim of this study was to clarify the background factors of FLD and NAFLD, and the influence of *H. pylori* infection on these diseases.

## Methods

### Study subjects

This was a cross-sectional study of Japanese asymptomatic adults who received medical checkups at Kameda Medical Center Makuhari (Chiba-shi, Chiba, Japan) in 2010, and voluntarily consented to entry into our study. This study was approved by the ethics committees of The University of Tokyo, and written forms of informed consent were obtained from all study participants according to the Declaration of Helsinki.

If the subject had health checkups twice in 2010, the former data was used. Criteria for exclusion were age less than 20 years, insufficient data, or poor answers to the questionnaire.

### Questionnaires

A detailed questionnaire including inquiries about upper gastrointestinal tract-related symptoms [44-46], medical history, family history, lifestyle factors, etc. was filled out by all the participants. This questionnaire has already been used and validated in several previous reports [31,46-49]. Answers filled out by the participants were carefully checked by the nursing staff before being recorded into our study database. The questionnaire included five yes-no questions regarding regular intake of anti-cholesterol drugs, anti-hypertensive drugs, anti-diabetic drugs and corticosteroids, and history of gastrectomy. We additionally graded alcohol intake frequency on a 5-grade scale (never, rarely, sometimes, almost daily, and daily per week). In this analysis, “never” and “rarely” are regarded as non-drinker, “sometimes” is regarded as occasional-drinker, and “almost daily” and “daily” are regarded as daily-drinker. As for the amount of alcohol intake, we defined one drink unit is equivalent to a 12-ounce beer, a 4-ounce glass of wine, or a 1-ounce shot of hard liquor. And we also categorized the subjects into four groups according to: less than two units at a time, two to three units at a time, three to four units at a time and more than four units at a time. We further categorized smoking habit into three groups, current smoking (current-smoker), past habitual smoking (former-smoker), and lifelong nonsmoking (never-smoker).

### Diagnosis of fatty liver disease (FLD)

Fatty liver disease (FLD) was diagnosed by abdominal ultrasonography. Routine ultrasonography evaluation of six intra-abdominal organs (liver, gallbladder, pancreas, kidneys, spleen, and abdominal aorta) was performed by well-trained operators. Characteristic findings of fatty liver are as follows; i) an increase of liver brightness, ii) an increase of hepato-renal echo contrast, iii) deep attenuation of hepatic echo, iv) existence of intra-hepatic vascular blurring, v) existence of focal hypoechoic lesion, and vi) existence of borderline blurring between liver and gallbladder, or right kidney. Fatty liver was diagnosed when the ultrasonographic findings satisfied both i) and ii) in addition to at least one of the findings between iii) to vi) [50]. The diagnosis was double-checked by the operators and gastroenterologists.

### Definition of non-alcoholic fatty liver disease (NAFLD)

NAFLD was defined according to the guideline published from AASLD (American Association for the Study of Liver Diseases), ACG (American College of Gastroenterology), and AGA (American Gastroenterological Association) in 2012 [7]. In this present study, we defined NAFLD according to the characteristic findings as follows; i) with evidence of fatty liver by ultrasonography (see above), and ii) with no causes for secondary hepatic fat accumulation including any viral hepatitis and steatogenic medication. With regard to alcohol intake, more than 21 drink units per week in males and more than 14 drink units per week in females were widely recognized as significant alcohol intake. To exclude alcohol influence strictly, we omitted all occasional-drinkers, all daily-drinkers, and Non-drinkers who occasionally drunk more than four units at one time.

### Evaluation of serum anti-helicobacter pylori antibody

Serum anti-*H. pylori* antibody was measured using a commercial EIA kit (E-plate “EIKEN” *H. pylori* antibody, EIKEN Chemical Co Ltd, Tokyo, Japan) as we have previously reported [48]. According to the manufacturer’s instruction, an antibody titer above 10 U/ml was considered as *H. pylori*-positive. We omitted individuals who had histories of eradications of *H. pylori* from analysis.

### Definition of metabolic syndrome (MS)

The definition of MS was based on the Japanese criteria published in 2005 [51]. The diagnostic criteria is visceral obesity in combination with any two of the following three standards; i) systolic blood pressure (SBP) greater than 130 mmHg and/or diastolic blood pressure (DBP) greater than 85 mmHg, ii) triglyceride (TG) greater than 150 mg/dL and/or high-density lipoprotein (HDL) cholesterol less than 40 mg/dL, iii) fasting blood sugar (FBS) greater than 110 mg/dL. Visceral obesity was defined as a waist girth of at least 85 cm in male and at least 90 cm in female.

### Statistical analyses

We analyzed data of female and male separately. We used JMP11 software (SAS Institute Japan) for statistical analyses. In univariate analyses, odds ratios and 95% confidence intervals were calculated and a *p* value of <0.01 was considered to indicate statistical significance. Following continuous variables were compared using the Welch’s t test or Wilcoxon’s rank-sum test and following categorical variables were compared using the Fisher’s exact test as appropriate for fatty liver status: age (continuous data), height (continuous data), weight (continuous data), BMI (body mass index, continuous data), AST (serum aspartate aminotransferase, continuous data), ALT (serum alanine aminotransferase, continuous data), GGT (gamma-glutamyl transpeptidase, continuous data), T-Bil (total bilirubin, continuous data), ALB (serum albumin, continuous data), PLT (platelet, continuous data), TC (total cholesterol, continuous data), HDL-C (high-density lipoprotein cholesterol, continuous data), LDL-C (low-density lipoprotein cholesterol, continuous data), TG (triglyceride, continuous data), HbA1c (continuous data), FBS (fasting blood sugar, continuous data), SBP (systolic blood pressure, continuous data), DBP (diastolic blood pressure, continuous data), Waist girth (continuous data), presence of MS (metabolic syndrome, categorical data), habit of drinking (categorical data), habit of smoking (categorical data), and *H. pylori* infection status (anti-*H. pylori* antibody, categorical data). Among the continuous variables, AST, ALT, GGT and TG were compared by Wilcoxon’s rank-sum test and the other continuous variables were compared by Welch’s t test.

In multivariate analysis, standardized coefficient and standard error of each variable for FLD and NAFLD were calculated by the regularized logistic regression via the elastic net to avoid the correlations between each variable and a *p* value of <0.01 was considered to indicate statistical significance.

## Results

### Participants

**Figure 1:**
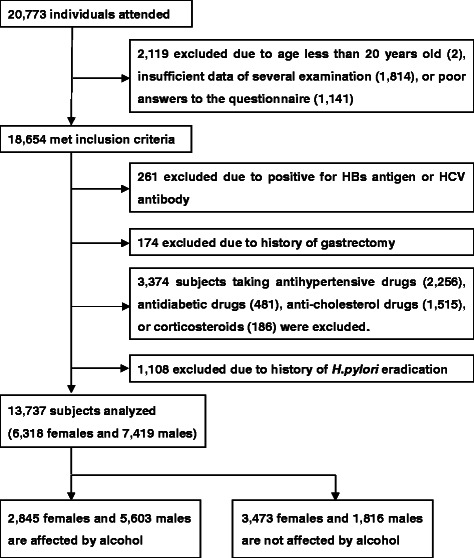
**Of the 20**,**773 asymptomatic adults attended**, **13**,**737 subjects were mainly analyzed in our present study.** The subcategory of 5,289 subjects who were not affected by alcohol was also analyzed. Each figure presenting excluded patients was overlapping at the same box.

Of the 20,773 subjects who participated in this study, we excluded 2,119 subjects due to age less than 20 years old (2), insufficient data of several examination including ultrasonography and anti-*H. pylori* antibody (1,814), or poor answers to the questionnaire (1,141). The 2,119 excluded subjects had the same background characteristics as the included 18,654 subjects (Additional file 1: Table S1). Of the 18,654 subjects who met inclusion criteria (Figure 1), we further excluded 3,809 subjects who were positive for HBsAg and/or HCVAb (261), who had a history of gastrectomy (174), and who took anti-hypertensive drugs (2,256), anti-diabetic drugs (481), anti-cholesterol drugs (1,515), or corticosteroids (186), since these factors might affect fatty liver status and/or some essential laboratory tests [52,53]. Finally, we further excluded 1,108 subjects who had a history of *H. pylori* eradication.

The primary study population of 13,737 subjects was comprised of 6,318 females and 7,419 males. Among them, fatty liver (FLD) was detected in 1,456 of 6,318 females (23.0%) and 3,498 of 7,419 males (47.1%). We also analyzed specific subjects who drank very little or no alcohol to remove the effects of alcohol intake to fatty liver status: after excluding all occasional-drinkers, all daily-drinkers, and non-drinkers who drank more than four units at a time, the residual 5,289 subjects (3,473 females and 1,816 males) were analyzed. Among them, fatty liver (i.e., NAFLD) was detected in 881 of 3,473 females (25.4%) and 921 of 1,816 males (50.7%).

### Associated factors for fatty liver disease (FLD) based on the univariate analysis

We analyzed 19 continuous variables and 4 categorized variables and their association with fatty liver disease (Table 1, Table 2). Among the 23 examined factors, age, weight, BMI, AST, ALT, GGT, T-Bil, PLT, TC, HDL-C, LDL-C, TG, HbA1c, FBS, SBP, DBP, waist girth, presence of MS and drinking habit were statistically significant background factors in both genders. Height, ALB, smoking habit and *H. pylori* infection status were statistically significant background factors in one gender.

**Table 1:** **Characteristics of the 13**,**737 subjects** (**6**,**318 females and 7**,**419 males**) **focusing on the presence of fatty liver disease** (**FLD**) **and its association with 19 continuous variables**

ᅟ	Female			Male		
Variables	FLD(N = 1456)	non-FLD(N = 4862)	*p*value	FLD(N = 3498)	non-FLD(N = 3921)	*p*value
Age (years old)	50.3 ± 7.9	46.9 ± 8.6	<0.0001*	48.7 ± 8.4	48.1 ± 9.5	0.0012*
Height (cm)	157.3 ± 5.6	158.2 ± 5.4	<0.0001*	170.8 ± 5.8	171.0 ± 5.9	0.0717
Weight (kg)	61.8 ± 9.6	51.4 ± 6.2	<0.0001*	72.8 ± 9.7	64.6 ± 7.8	<0.0001*
BMI (kg/m^2^)	25.0 ± 3.6	20.5 ± 2.2	<0.0001*	24.9 ± 2.8	22.1 ± 2.2	<0.0001*
AST (IU/l)	21.2 ± 8.9	19.0 ± 5.4	<0.0001*	24.6 ± 10.0	21.3 ± 17.1	<0.0001*
ALT (IU/l)	23.0 ± 16.7	16.0 ± 7.6	<0.0001*	33.5 ± 22.6	21.6 ± 15.9	<0.0001*
GGT (IU/l)	28.3 ± 27.8	19.3 ± 17.3	<0.0001*	55.6 ± 58.2	38.8 ± 38.3	<0.0001*
T-Bil (mg/dl)	0.71 ± 0.27	0.76 ± 0.27	<0.0001*	0.87 ± 0.35	0.90 ± 0.37	0.0045*
ALB (g/dl)	4.26 ± 0.21	4.25 ± 0.21	0.1028	4.40 ± 0.22	4.36 ± 0.22	<0.0001*
PLT (10^4^/μl)	26.1 ± 5.9	23.7 ± 5.4	<0.0001*	23.9 ± 4.9	22.9 ± 4.7	<0.0001*
TC (mg/dl)	214.0 ± 34.1	198.8 ± 33.0	<0.0001*	207.7 ± 32.5	195.4 ± 30.5	<0.0001*
HDL-C (mg/dl)	64.7 ± 14.2	75.8 ± 15.4	<0.0001*	54.1 ± 12.7	63.8 ± 15.2	<0.0001*
LDL-C (mg/dl)	135.0 ± 32.3	114.2 ± 29.9	<0.0001*	134.8 ± 30.2	119.4 ± 28.9	<0.0001*
TG (mg/dl)	107.2 ± 80.8	68.2 ± 32.0	<0.0001*	151.3 ± 97.6	98.7 ± 62.6	<0.0001*
HbA1c (%)	5.52 ± 0.56	5.30 ± 0.33	<0.0001*	5.50 ± 0.62	5.29 ± 0.37	<0.0001*
FBS (mg/dl)	94.9 ± 15.2	88.3 ± 8.5	<0.0001*	100.1 ± 16.4	94.1 ± 10.0	<0.0001*
SBP (mmHg)	118.8 ± 16.5	107.7 ± 14.5	<0.0001*	121.4 ± 15.0	115.4 ± 14.7	<0.0001*
DBP (mmHg)	73.9 ± 10.5	67.2 ± 9.6	<0.0001*	77.0 ± 10.1	73.0 ± 9.8	<0.0001*
Waist (cm)	87.2 ± 8.6	75.7 ± 6.5	<0.0001*	87.4 ± 7.1	79.8 ± 6.4	<0.0001*

*FLD*: fatty liver disease. Data show mean ± *SD* (standard deviation) of each variable. By applying the Welch’s t test or Wilcoxon analysis, *p* values were calculated. The level of significance was set below 0.01 (*).

**Table 2:** **Characteristics of the 13**,**737 subjects** (**6**,**318 females and 7**,**419 males**) **focusing on the presence of fatty liver disease** (**FLD**) **and its association with four categorized variables**

ᅟ	Female			Male		
Variables	FLD(N = 1456)	non-FLD(N = 4862)	*p*value	FLD(N = 3498)	non-FLD(N = 3921)	*p*value
MS	ᅟ	ᅟ	<0.0001*	ᅟ	ᅟ	<0.0001*
Non-MS	1391 (22.3 %)	4860 (77.6 %)	ᅟ	2945 (43.5 %)	3832 (56.5 %)	ᅟ
MS	65 (97.0 %)	2 (3.0 %)		553 (86.1 %)	89 (13.9 %)	ᅟ
Alcohol	ᅟ	ᅟ	<0.0001*	ᅟ	ᅟ	<0.0001*
Non-drinker	884 (25.3 %)	2604 (74.7 %)		951 (50.8 %)	923 (49.3 %)	ᅟ
Occasional-drinking	368 (20.2 %)	1456 (79.8 %)	ᅟ	1114 (49.9 %)	1120 (50.1 %)	ᅟ
Daily-drinker	204 (20.3 %)	802 (79.7 %)	ᅟ	1433 (43.3 %)	1878 (56.7 %)	ᅟ
Smoking	ᅟ	ᅟ	0.0467	ᅟ	ᅟ	0.0045*
Current-smoker	123 (27.8 %)	319 (72.2 %)		1116 (49.3 %)	1147 (50.7 %)	
Former-smoker	174 (23.2 %)	577 (76.8 %)	ᅟ	1317 (47.6 %)	1450 (52.4 %)	ᅟ
Never-smoker	1159 (22.6 %)	3966 (77.4 %)	ᅟ	1065 (44.6 %)	1324 (55.4 %)	ᅟ
*H. pylori* Ab	ᅟ	ᅟ	<0.0001*	ᅟ	ᅟ	0.6446
Negative	1003 (21.5 %)	3665 (78.5 %)	ᅟ	2474 (47.0 %)	2793 (53.0 %)	ᅟ
Positive	453 (27.5 %)	1197 (72.6 %)	ᅟ	1024 (47.6 %)	1128 (52.4 %)	ᅟ

*FLD*: fatty liver disease, *MS*: metabolic syndrome, *Ab*: antibody. By applying the Fisher’s exact test, *p* values were calculated. The level of significance was set below 0.01 (*).

According to the results of univariate analyses and multicollinearity (0.8957 in females and 0.8723 in males between weight and BMI, 0.8521 in females and 0.7791 in males between AST and ALT), we excluded height, weight, AST and smoking habit from the next multivariate analysis. We adopted MS as a representative of waist girth, TC, HDL-C, LDL-C, TG, FBS, HbA1c, SBP and DBP. Consequently, we focused on the following nine variables for multivariate analysis: age, BMI, ALT, GGT, T-Bil, PLT, MS, drinking habit, and *H. pylori* infection status.

### Associated factors for fatty liver disease (FLD) based on the multivariate analysis

As shown in Table 3, the generalized regression analysis demonstrated that BMI, ALT, age, and PLT were positively associated with FLD in both genders, and MS was positively associated only in males. Daily-drinking habit was negatively associated with FLD only in male. In contrast, GTT, T-Bil, and *H. pylori* infection status were not associated with the presence of FLD.

**Table 3:** **Multivariate analyses evaluating association between the presence of fatty liver disease** (**FLD**) **and 9 selected variables among the 13**,**737 subjects** (**6**,**318 females and 7**,**419 males**).

ᅟ	Female			Male		
Variables	Standardized coefficient	Standard error	*p*value	Standardized coefficient	Standard error	*p*value
BMI	143.5	4.8	<0.0001*	102.5	3.7	<0.0001*
ALT	25.8	5.1	<0.0001*	75.7	13.1	<0.0001*
Age	34.3	3.5	<0.0001*	17.2	2.8	<0.0001*
PLT	17.8	3.1	<0.0001*	15.2	2.6	<0.0001*
Alcohol	ᅟ	ᅟ	ᅟ	ᅟ	ᅟ	ᅟ
Occasional-drinker	−8.1	3.7	0.0270	−5.9	2.8	0.0371
Daily-drinker	8.5	3.7	0.0209	−8.4	2.9	0.0040*
MS	ᅟ	ᅟ	ᅟ	ᅟ	ᅟ	ᅟ
Positive	10.2	5.9	0.0804	16.4	3.2	<0.0001*
GGT	5.3	3.1	0.0919	4.0	4.1	0.3399
T-Bil	−3.1	3.3	0.3386	1.4	2.4	0.5555
*H. pylori*	ᅟ	ᅟ	ᅟ	ᅟ	ᅟ	ᅟ
Positive	1.0	3.1	0.7558	−2.8	2.5	0.2642

*MS*: metabolic syndrome, *C.I*.: confidence interval, *Ab*: antibody, Using the logistic regression analysis, *p* values were calculated. The level of significance was set below 0.01 (*).

### Associated factors for non-alcoholic fatty liver disease (NAFLD) based on the univariate analysis

Furthermore, we analyzed the 5,289 subjects free from alcohol influence to evaluate the relationship between NAFLD and background factors including *H. pylori* infection status. We analyzed 19 continuous variables and 3 categorized variables and their association with fatty liver disease (Tables 4 and 5). Among the 22 examined factors, weight, BMI, AST, ALT, GGT, PLT, TC, HDL-C, TG, HbA1c, FBS, SBP, DBP, waist girth, and MS were statistically significant in both genders. Age, height, T-Bil, ALB, and smoking habit were statistically significant in only one gender. However, regardless of gender, *H. pylori* infection status was not associated with the presence of NAFLD.

**Table 4:** **Characteristics of the alcohol**-**free 5**,**289 subjects** (**3**,**473 females and 1**,**816 males**) **focusing on the presence of fatty liver** (**NAFLD**) **and its association with 19 continuous variables**

ᅟ	Female			Male		
Variables	NAFLD(N = 881)	non-NAFLD(N = 2592)	*p*value	NAFLD(N = 921)	non-NAFLD(N = 895)	*p*value
Age (years old)	50.8 ± 8.1	47.6 ± 8.9	<0.0001*	47.6 ± 8.6	47.4 ± 10.1	0.5126
Height (cm)	156.9 ± 5.4	157.8 ± 5.5	<0.0001*	170.7 ± 5.8	170.9 ± 6.0	0.4956
Weight (kg)	61.8 ± 9.8	51.2 ± 6.3	<0.0001*	72.8 ± 10.0	63.8 ± 8.0	<0.0001*
BMI (kg/m^2^)	25.1 ± 3.7	20.5 ± 2.3	<0.0001*	25.0 ± 3.0	21.8 ± 2.3	<0.0001*
AST (IU/l)	21.4 ± 9.2	19.0 ± 5.4	<0.0001*	23.8 ± 10.6	20.0 ± 5.3	<0.0001*
ALT (IU/l)	23.5 ± 17.7	16.2 ± 7.8	<0.0001*	35.1 ± 30.7	21.3 ± 9.4	<0.0001*
GGT (IU/l)	26.4 ± 24.6	18.2 ± 14.8	<0.0001*	40.5 ± 41.0	26.6 ± 22.4	<0.0001*
T-Bil (mg/dl)	0.71 ± 0.26	0.74 ± 0.26	<0.0004*	0.85 ± 0.36	0.86 ± 0.37	0.5524
ALB (g/dl)	4.26 ± 0.22	4.24 ± 0.21	0.0148	4.41 ± 0.23	4.35 ± 0.22	<0.0001*
PLT (10^4^/μl)	26.1 ± 6.0	23.7 ± 5.6	<0.0001*	24.7 ± 5.1	22.9 ± 4.9	<0.0001*
TC (mg/dl)	213.2 ± 33.4	201.2 ± 33.6	<0.0001*	207.4 ± 32.9	192.6 ± 29.9	<0.0001*
HDL-C (mg/dl)	62.9 ± 14.0	74.1 ± 15.0	<0.0001*	50.5 ± 10.7	59.5 ± 13.8	<0.0001*
LDL-C (mg/dl)	136.0 ± 31.8	118.2 ± 30.3	<0.0001*	138.6 ± 29.5	121.3 ± 27.8	<0.0001*
TG (mg/dl)	107.8 ± 71.0	69.1 ± 31.2	<0.0001*	144.1 ± 92.0	91.6 ± 47.7	<0.0001*
HbA1c (%)	5.55 ± 0.54	5.34 ± 0.35	<0.0001*	5.51 ± 0.63	5.31 ± 0.40	<0.0001*
FBS (mg/dl)	94.5 ± 14.7	88.1 ± 9.1	<0.0001*	98.4 ± 17.0	92.2 ± 8.5	<0.0001*
SBP (mmHg)	119.4 ± 16.8	107.7 ± 14.4	<0.0001*	118.6 ± 14.5	111.2 ± 13.3	<0.0001*
DBP (mmHg)	74.2 ± 10.6	66.9 ± 9.5	<0.0001*	74.9 ± 9.4	70.0 ± 8.8	<0.0001*
Waist (cm)	87.3 ± 8.7	75.7 ± 6.5	<0.0001*	87.3 ± 7.5	78.6 ± 6.6	<0.0001*

*NAFLD*: non-alcoholic fatty liver disease. Data show mean ± *SD* (standard deviation) of each variable. By applying the Welch’s t test or Wilcoxon analysis, *p* values were calculated. The level of significance was set below 0.01 (*).

**Table 5:** **Characteristics of the alcohol**-**free 5**,**289 subjects** (**3**,**473 females and 1**,**816 males**) **focusing on the presence of fatty liver** (**NAFLD**) **and its association with three categorized variables**

ᅟ	Female			Male		
Variables	NAFLD(N = 881)	non-NAFLD(N = 2592)	*p*value	NAFLD(N = 921)	non-NAFLD(N = 895)	*p*value
MS	ᅟ	ᅟ	<0.0001*	ᅟ	ᅟ	<0.0001*
Non-MS	838 (24.5 %)	2590 (75.6 %)		793 (47.2 %)	886 (52.8 %)	ᅟ
MS	43 (95.6 %)	2 (4.4 %)	ᅟ	128 (93.4 %)	9 (6.6 %)	ᅟ
Smoking	ᅟ	ᅟ	0.3913	ᅟ	ᅟ	0.0033*
Current-smoker	54 (29.4 %)	130 (70.7 %)		289 (54.4 %)	242 (45.6 %)	ᅟ
Former-smoker	71 (24.0 %)	225 (76.0 %)	ᅟ	284 (53.7 %)	245 (46.3 %)	ᅟ
Never-smoker	756 (25.3 %)	2237 (74.7 %)	ᅟ	348 (46.0 %)	408 (54.0 %)	ᅟ
*H. pylori* Ab	ᅟ	ᅟ	0.0145	ᅟ	ᅟ	0.8742
Negative	610 (24.2 %)	1907 (75.8 %)	ᅟ	669 (50.6 %)	654 (49.4 %)	ᅟ
Positive	271 (28.4 %)	685 (71.7 %)	ᅟ	252 (51.1 %)	241 (48.9 %)	ᅟ

*NAFLD*: non-alcoholic fatty liver disease, *MS*: metabolic syndrome, *Ab*: antibody. By applying the Fisher’s exact test, *p* values were calculated. The level of significance was set below 0.01 (*).

According to the results of univariate analyses and multicollinearity (0.9024 in female and 0.8841 in male between weight and BMI, 0.8626 in female and 0.8440 in male between AST and ALT), we excluded height, weight, AST, T-Bil, and smoking habit from the next multivariate analysis. We adopted MS as a representative of waist girth, TC, HDL-C, LDL-C, TG, FBS, HbA1c, SBP and DBP. Consequently, we chose the following seven variables for multivariate analysis: Age, BMI, ALT, GGT, PLT, MS, and *H. pylori* infection status.

### Associated factors for non-alcoholic fatty liver disease (NAFLD) based on the multivariate analysis

As shown in Table 6, the generalized regression analysis displayed that BMI, ALT, and PLT were positively associated with the presence of NAFLD. Age was positively associated with NAFLD only in females, whereas MS was positively associated only in males. Like FLD, *H. pylori* infection status did not show significant association with NAFLD, regardless of gender.

**Table 6:** **Multivariate analysis evaluating association between NAFLD and 7 selected variables among the non**-**drinking 5**,**289 subjects** (**3**,**473 females and 1**,**816 males**)

ᅟ	Female			Male		
Variables	Standardized Coefficient	Standard error	*p*value	Standardized Coefficient	Standard error	*p*value
BMI	113.3	4.9	<0.0001*	55.3	3.7	<0.0001*
ALT	21.6	6.1	0.0004*	53.8	6.5	<0.0001*
Age	25.0	3.5	<0.0001*	3.7	2.6	0.1607
PLT	13.8	3.0	<0.0001*	11.8	2.5	<0.0001*
MS	ᅟ	ᅟ	ᅟ	ᅟ	ᅟ	ᅟ
Positive	5.9	5.0	0.2354	11.6	4.5	0.0092*
GGT	2.3	3.4	0.4975	0.24	3.9	0.9508
*H. pylori*	ᅟ	ᅟ	ᅟ	ᅟ	ᅟ	ᅟ
Positive	−1.2	3.1	0.7017	−1.8	2.6	0.4764

*MS*: metabolic syndrome, *Ab*: antibody, *C.I*.: confidence interval. Using the logistic regression analysis, *p* values were calculated. The level of significance was set below 0.01 (*).

## Discussion

### Background factors for FLD

As for FLD, BMI, ALT, age and PLT were positively associated with the presence of FLD in both genders, which is concurrent with the recent study report from Japan [54].

Although alcohol intake has been regarded as a main cause for fatty liver for a long time [1,55-58], our result did not demonstrate a significant positive association between the frequency of alcohol intake and the presence of fatty liver. Occasional alcohol intake tended to be negatively associated with fatty liver in both genders, similarly to a recent report [8]. (Table 3). There is a possibility that the negative association of alcohol intake to FLD in daily-drinking males is attributed to the design of this study, since the evaluation of amounts of alcohol intake was not quantitatively accurate. To validate our result; the association between alcohol and fatty liver may not be as significant as previously reported, more detailed information of alcohol intake and careful setup of the study cohort will be needed in future studies.

### Background factors for NAFLD

As for NAFLD, BMI, ALT, and PLT showed positive association. Unlike FLD, age was positively associated with the presence of NAFLD only in females (Table 6). Female gender, age, diabetes mellitus, hyperinsulinaemia, obesity and hypertriglyceridaemia have been regarded as traditional risk factors for NAFLD [3,59-61]. Our results denoted the same tendency of these previous reports, but MS wasn’t associated with the presence of NAFLD in females.

### Association between H. pylori and FLD including NAFLD

Concerning *H. pylori* infection, neither FLD nor NAFLD displayed a significant association in multivariate analysis regardless of gender, though *H. pylori* infection showed significant association in univariate analysis in FLD in female. This suggests that upper gastro-intestinal environment caused by chronic *H. pylori* infection has no or marginal influence on the development of fatty liver. These results are consistent with some previous reports [35,36].

### Limitations and future prospects

The first limitation of our study is the study design itself (i.e., cross-sectional study). A single point analysis cannot obtain accurate results, since fatty liver is thought to emerge as a consequence of several risk factors over a number of years. The second limitation is reliability of ultrasonography as a diagnostic tool for NAFLD. Diagnosis by ultrasonography has inevitable limitations due to low sensitivity for mild steatosis, inability to differentiate mild fibrosis from steatosis, and inaccurate quantification of fatty infiltration [62]. And more, interobserver and intraobserver variability in the sonographic assessment of fatty liver are well known [63]. Nevertheless, these shortcomings were thought to be minimal, because well-trained operators and gastroenterologists in a single hospital diagnosed fatty liver with prescribed findings written above in this study. In fact, we have already reported some study results based on our ultrasonography-based diagnoses [31,64]. The third limitation is the diagnostic accuracy of *H. pylori* infection based on serology. Though urea breath test (UBT) is superior to the serology test, the serology test is used due to its non-invasiveness and cost-effectiveness in our medical check up, which routinely has blood drawing. The accuracy of serology test in diagnosing *H. pylori* infection in this study was acceptable, since our recent study using the same study population showed that 97.8% (1,638 of 1,674) of the subjects with sero-positivity of *H. pylori* had chronic atrophic gastritis[49]. The fourth limitation is the small number of female participants. We inferred that undetected association between MS and FLD including NAFLD might be due to the small number of female subjects who met MS criteria.

For the future prospects, we will follow this cohort to reveal the long-term effect of various associated factors upon FLD and NAFLD, such as developing NASH, liver cirrhosis, hepatocellular carcinoma, and so on.

## Conclusions

Body mass index (BMI), serum ALT, and platelet count were positively associated with the presence of fatty liver disease (FLD) and non-alcoholic fatty liver disease (NAFLD) in both genders. On the other hands, *Helicobacter pylori* infection was not associated with either FLD or NAFLD.

## Additional file

Additional file 1: Table S1. Background characteristics of the included 18,654 subjects and the excluded 2,119 subjects.

## Abbreviations

FLD: Fatty liver disease
NAFLD: Non-alcoholic fatty liver disease
AFLD: Alcoholic fatty liver disease
HCC: Hepatocellular carcinoma
*H. pylori*: *Helicobacter pylori*
BMI: Body mass index
AST: Aspartate aminotransferase
ALT: Alanine aminotransferase
GGT: Gamma-glutamyl transpeptidase
T-Bil: Total bilirubin
ALB: Albumin
PLT: Platelet
TC: Total cholesterol
HDL-C: High-density lipoprotein
LDL-C: Low-density lipoprotein cholesterol
TG: Triglyceride
FBS: Fasting blood sugar
SBP: Systolic blood pressure
DBP: Diastolic blood pressure
MS: Metabolic syndrome
