# Supplementary material for: Helicobacter pylori infection is not associated with fatty liver disease including non-alcoholic fatty liver disease: a large-scale cross-sectional study in Japan
Source: BMC Gastroenterol. 2015 Feb 19;15:25. doi: 10.1186/s12876-015-0247-9 (PMC4349671; doi:10.1186/s12876-015-0247-9)
Supplement: Additional file 1: Table S1. — Background characteristics of the included 18,654 subjects and the excluded 2,119 subjects. [file 12876_2015_247_MOESM1_ESM.docx]

Supplementary Table 1. Background characteristics of the included 18,654 subjects and the excluded 2,119 subjects.

| Variables | Included (N=18,654) | Excluded (N=2,119) |
| --- | --- | --- |
| Age (years old) | 49.8 ± 9.2 | 53.1 ± 11.3 |
| BMI (kg/m^2^) | 22.9 ± 3.3 | 22.7 ± 3.4 |
| Sex |  |  |
| Female | 7861 (42.1 %) | 997 (47.5 %) |
| Male | 10793 (57.9 %) | 1102 (52.5 %) |
| *H. pylori* |  |  |
| Negative | 13528 (72.5 %) | 936 (71.9 %) |
| Positive | 5126 (27.5 %) | 365 (28.1 %) |
| FLD |  |  |
| Negative | 11052 (59.2 %) | 1139 (60.8 %) |
| Positive | 7602 (40.8 %) | 734 (39.2 %) |

Data show mean ± SD (standard deviation) of each continuous variable.
